# Nested case–control sampling without replacement

**DOI:** 10.1007/s10985-024-09633-y

**Published:** 2024-09-05

**Authors:** Yei Eun Shin, Takumi Saegusa

**Affiliations:** 1 Seoul National University, Seoul, Korea; 2 University of Maryland, College Park, Maryland United States

**Keywords:** Conditional logistic regression, Inverse probability weighting, Nested case–control designs, Pseudo-partial likelihood, Sampling distribution

## Abstract

Nested case–control design (NCC) is a cost-effective outcome-dependent design in epidemiology that collects all cases and a fixed number of controls at the time of case diagnosis from a large cohort. Due to inefficiency relative to full cohort studies, previous research developed various estimation methodologies but changing designs in the formulation of risk sets was considered only in view of potential bias in the partial likelihood estimation. In this paper, we study a modified design that excludes previously selected controls from risk sets in view of efficiency improvement as well as bias. To this end, we extend the inverse probability weighting method of Samuelsen which was shown to outperform the partial likelihood estimator in the standard setting. We develop its asymptotic theory and a variance estimation of both regression coefficients and the cumulative baseline hazard function that takes account of the complex feature of the modified sampling design. In addition to good finite sample performance of variance estimation, simulation studies show that the modified design with the proposed estimator is more efficient than the standard design. Examples are provided using data from NIH-AARP Diet and Health Cohort Study.

## Introduction

The nested case–control (NCC) design proposed by Thomas (1977) is an outcome-dependent cohort sampling design in epidemiology that selects controls for each case arising in a cohort from individuals at risk at the time of case diagnosis. Compared to cohort studies, NCC studies significantly reduce the cost of data collection and save the resource for error checking by measuring expensive covariate values only for cases who experienced failure events and the sampled controls. A commonly used statistical model to analyze the NCC data is the Cox proportional hazards model (Cox 1972). The standard method for estimating regression coefficients (relative hazards) in the NCC design is to maximize the partial likelihood (Thomas 1977; Oakes 1981) which coincides with the conditional logistic likelihood for matched case–control studies (Breslow et al. 1978).

A notable feature of the NCC design is to potentially have duplicated individuals as controls and cases. In the standard setting, the fixed number of controls are selected by simple random sampling from the risk set at each failure time, consisting of uncensored individuals who have not yet experienced the failure event of interest. Because some individuals belong to multiple risk sets at different event times, they can be selected as controls more than once, and those sampled controls may experience the failure event later. Although sampling the same individuals may reduce the cost of data collection especially when covariates are time-independent, it would fail to efficiently obtain information from the entire cohort. Instead, we hypothesize that selecting new controls who are not previously sampled is expected to improve efficiency. This modification of sampling controls without replacement is not difficult to implement since one can simply exclude previously sampled individuals from the risk sets afterwards. Also, the cost is more or less the same as the standard design particularly for time-varying covariates.

The NCC study is less efficient than the cohort study because it uses the partial information of the entire cohort. There are many research to overcome the issue of inefficiency of the NCC studies. Samuelsen (1997) proposed the inverse probability weighting (IPW) approach and showed its superior performance over the usual partial likelihood estimator (Borgan and Keogh 2015). Other researchers improved the efficiency of NCC studies by incorporating additional data available in the cohort via full likelihood estimation (Saarela et al. 2008), weight calibration (Rivera and Lumley 2016; Shin et al. 2020, 2021) or multiple imputation (Keogh et al. 2018; Borgan and Keogh 2015). However, one important aspect which previous research paid much less attention to is that several variants of the NCC design are available depending on how to select controls from risk sets. In this paper, we study an alternative NCC design with an emphasis on efficiency gain rather than proposing a new method in the standard setting.

Various implementations of alternative NCC designs have been already studied in the literature. These designs differ, for example, by considering whether a case itself can serve as its own control, whether a control who later develops a disease should be excluded, or whether a control who later becomes censored should be excluded (Lubin and Gail 1984). Others considered the size of risk sets depending on the sparsity of stratification and the choice of time scale (age or time on study; that is, open or closed cohort) (Robins et al. 1986). The main focus of these studies is to evaluate the potential bias of the partial likelihood estimator due to changes in sampling schemes. However, the issue of efficiency improvement nor Samuelsen’s more efficient estimator (Samuelsen 1997) are outside of their scope.

In this paper, we modify the NCC design to improve estimation efficiency such that individuals are selected as controls at most once and allow duplication only when previously selected controls later become cases. We study the bias and efficiency of risk estimates in this modified design and identify major factors that improves efficiency over the standard design through extensive simulation studies. In addition to the partial likelihood estimator, we extend a more efficient inverse probability weighted estimator of Samuelsen (1997), which breaks the matching structure of cases and controls at the time of sampling and pooled all selected controls and cases as a subset of cohort. The risk set at event time *t* in this estimator contains not only controls sampled at *t* but also controls sampled at other times as long as they are not censored but at risk at *t* so that this expansion makes estimation more efficient. However, because it may cause selection bias as individuals with longer survival time are more likely to be in risk sets at many different event times, we also present bias correction of inclusion probability weights for the modified design.

In addition to regression coefficients in the Cox model, we study the estimation of the cumulative baseline hazard function. Efficient estimation of both regression coefficients and baseline hazard function is crucial in producing reliable estimates of the predictions of exposure-specific individual risks (Ganna et al. 2012; Shin et al. 2020). We consider the Breslow-type estimator for the baseline hazard function (Breslow 1972) which can be weighted with a subsample of cohort. For the estimation in line with the partial likelihood estimator, we study the Langholz–Borgan weights proposed in the standard NCC sampling (Langholz and Borgan 1997). For the IPW approach, we adopt the inverse of the same inclusion probabilities as those in the estimation of regression coefficients developed in this paper.

## Standard nested case–control sampling design

### Notations

Denote $T_i$ and $C_i$ as a survival time and a censoring time respectively for subject $i =1, \ldots , N$. Under right censoring, we only observe the minimum $X_i=\min (T_i, C_i)$ of $T_i$ and $C_i$. The risk set at *t* is $\mathcal {R}(t) = \{i \mid Y_{it}=1 \}$, where $Y_{it}=1$ if subject *i* is at risk at *t* or 0 otherwise. We consider the time-on-study scale so that $Y_{it}=I(x_i\ge t)$, where *t* is a time on study, $x_i$ is the time on study for subject *i*, and assume there is no ties in observed case times for simplicity. Let $\text{N}_i(t) = I(T_i\le t)$ denote a binary counting process that jumps when subject *i* experiences a failure and $\text{dN}_i(t)=\text{N}_i(t)-\text{N}_i(t-)$ indicate the increment of $\text{N}_i$ at time *t*, where $\text{N}_i(t-) = \lim _{\epsilon \downarrow 0} \text{N}_i(t-\epsilon )$. We define $\delta _i = \text{dN}_i(x_i)$ as a case indicator whether subject *i* develops a case at $x_i$ (i.e., $\delta _i=1$ if $x_i=\min (t_i, c_i)=t_i$ and 0 otherwise). The hazard function is $\lambda (t)=\lim _{\epsilon \downarrow 0}\text {P}(t\le T<t+\epsilon \mid T\ge t)/\epsilon$, which is specified as $\lambda (t)=\lambda _0(t)\exp (\varvec{\beta }^\text {T}\varvec{Z})$ for the Cox proportional hazard model (Cox 1972) where $\lambda _0(t)$ is the baseline hazard function and $\varvec{\beta }$ is the vector of log-relative hazards associated with a covariate vector $\varvec{Z}$.

### Sampling scheme

The standard NCC design samples *m* controls every time a case develops during the follow-up period. Any selected control may be sampled again at future case times or may develop a case later. Without loss of generality, denote $t_k$ for $k=1, \ldots , D$ as time at which case *k* develops where $D=\sum _{i=1}^N\delta _i$ is the total number of cases in the full cohort. If controls were matched to case *k* based only on at-risk status at $t_k$, the sampling pool at $t_k$ is $\mathcal {R}_k\backslash \{k\}$ where $\mathcal {R}_k=\mathcal {R}(t_k)$ and $A\backslash B$ denotes the set *A* excluding *B*. If there were additional matching criteria other than at-risk status (e.g., gender, ethnicity, or residence), the sampling pool at $t_k$ is $(\mathcal {R}_k\cap \mathcal {M})\backslash \{k\}$ where $\mathcal {M}$ consists of subjects that meet those additional criteria. Hereafter we assume that controls are matched based only on at-risk status for simplicity.

### Conditional estimation on the matching structure

The partial likelihood estimator uses the matching structure of cases to controls via sampled risk sets. Let $\widetilde{\mathcal {R}}(t_k) = \{k, j_1, j_2, \ldots , j_m\}\subset \mathcal {R}(t_k)$ denote the sampled risk sets for $k=1,\ldots , D$ (i.e., matching strata) in which subject *k* develops a case at $t_k$ and $j_1, \ldots , j_m$ are indices of selected controls at $t_k$. The log-relative hazards $\varvec{\beta }$ is estimated by maximizing the partial likelihood (Thomas 1977; Prentice and Breslow 1978; Oakes 1981),

1 $$\begin{aligned} \widehat{\varvec{\beta }}_c = \arg \max _{\varvec{\beta }} \mathcal {L}_\text {c}(\varvec{\beta }) = \arg \max _{\varvec{\beta }} \prod _{i=1}^N\prod _{k=1}^D\Bigg \{\frac{\exp ({\varvec{\beta }}^\text {T}{\varvec{Z}}_i)}{\sum _{j\in \widetilde{\mathcal {R}}(t_k)} \exp ({\varvec{\beta }}^\text {T}{\varvec{Z}}_j)}\Bigg \}^{\text{dN}_i(t_k)}. \end{aligned}$$

The resulting estimates are equivalent to the estimates from conditional logistic regression (Breslow et al. 1978), which is a conditional analysis where each case is compared with its own control set. In other words, the conditional logistic regression is a logistic regression allowing a different constant for each stratum, $\widetilde{\mathcal {R}}(t_k)$, and has the conditional likelihood identical to (1). To estimate $\text{d}\Lambda _0(t)$, Langholz and Borgan (1997) proposed a weighted Breslow estimator

2 $$\begin{aligned} \text{d}\widehat{\Lambda }_0^c(t\mid \widehat{\varvec{\beta }}_c) = \frac{\sum _{i=1}^N\text{dN}_i(t)}{\sum _{i\in \widetilde{\mathcal {R}}(t)} \{|\mathcal {R}(t)|/|\widetilde{\mathcal {R}}(t)|\}\exp (\widehat{\varvec{\beta }}_c^\text {T}{\varvec{Z}}_i)}, \end{aligned}$$

where $|\widetilde{\mathcal {R}}(t)|=m+1$ (i.e., a case and *m* matched controls) at every case time $t = t_1,\ldots ,t_D$. The weight $|\mathcal {R}(t_k)|/|\widetilde{\mathcal {R}}(t_k)|$ is the ratio of the sizes of risk set in the full cohort to the sampled risk set at the case time $t_k$. With this weights, a sampled risk set $\widetilde{\mathcal {R}}(t)$ is weighted to represent a risk set $\mathcal {R}(t)$ in the entire cohort. Similar to the inference for the full cohort study, the variances of $\widehat{\varvec{\beta }}_c$ and $\widehat{\Lambda }_0^c$ may be estimated by the observed information matrix and the Taylor’s approximation, respectively [see also the Section 3 of Langholz and Borgan (1997)]. These estimated relative and baseline hazards can be used to estimate survival probability or absolute risk for a given set of covariates.

### Estimation from a pooled subset: inverse probability weighting

The IPW estimator expands a risk set to a pooled risk set $\overline{\mathcal {R}}(t) = \big \{j \in \cup _{k=1}^D \widetilde{\mathcal {R}}(t_k)\mid Y_{jt_k}=1\big \}$, which includes all cases and controls at risk at time $t_k$ by breaking their matching structure. Individuals in $\overline{\mathcal {R}}(t)$ are unique whether or not the subject is sampled more than once as a case and/or a control. Then $\varvec{\beta }$ is estimated by maximizing the pseudo-partial likelihood,

3 $$\begin{aligned} \widehat{\varvec{\beta }}_s = \arg \max _{\varvec{\beta }}\mathcal {L}_{ps}(\varvec{\beta }) = \arg \max _{\varvec{\beta }}\prod _{i=1}^N\prod _{k=1}^D\Bigg \{\frac{\exp ({\varvec{\beta }}^\text {T}{\varvec{Z}}_i)}{\sum _{j\in \overline{\mathcal {R}}(t_k)} w_j \exp ({\varvec{\beta }}^\text {T} {\varvec{Z}}_j)}\Bigg \}^{\text{dN}_i(t_k)}, \end{aligned}$$

which replaces the denominator of the conditional probabilities of the partial likelihood with the inclusion probability weighted sum where the weights are $w_i=1$ for $\delta _i=1$ (case) and $w_i=1/\pi _i$ for $\delta _i=0$ (non-case). Here, $\pi _i$ is the probability that subject *i* is ever sampled as a control (Samuelsen 1997). Details on $\pi$ will be presented in Sect. 3.3.1. The estimator of $\text{d}\Lambda _0(t)$ is similarly obtained as the weighted Breslow estimator as

4 $$\begin{aligned} \text{d}\widehat{\Lambda }_0^s(t\mid \widehat{\varvec{\beta }}_s) = \frac{\sum _{i=1}^N \text{dN}_i(t)}{\sum _{i\in \overline{\mathcal {R}}(t)} w_i \exp {(\widehat{\varvec{\beta }}_s^\text {T}{\varvec{Z}}_i})}. \end{aligned}$$

The variance of $\widehat{\varvec{\beta }}_s$ and $\widehat{\Lambda }_0^s$ can be estimated by the approximation of pseudo-information matrix using Taylor’s series [for more details, see the Section 3.3 of Samuelsen (1997) and the Section 4.2 of Shin et al. (2020)].

## Modified nested case–control sampling design without replacement

### Sampling scheme

In the standard NCC design, some subjects at risk at the multiple case times can be sampled more than once. It is inefficient to spend time and money for recruiting people who have already been selected before. For this reason, it is a common practice to modify the sampling scheme to select *m* controls at each $t_k$ from the sampling pool of at-risk individuals excluding units ever sampled (Corrales-Medina et al. 2015). In the modified NCC design, individuals once sampled are not replaced back to the future sampling pools and so have no chance to be sampled again later on. As a result, each unit can be selected as a control at most once. The sampling pool of the modified NCC design at $t_k$ is therefore $\mathcal {R}_k\backslash \{\mathcal {Q}_k\cup \{k\}\}$ where $\mathcal Q_{k}$ is a set of selected controls before $t_k$ with $\mathcal {Q}_1=\varnothing$. Note that some controls may be duplicated as cases even with the modified design because it is unknown at the time of sampling whether or when currently at-risk individuals experience the event of interest later in a prospective cohort study.

**Fig. 1:**
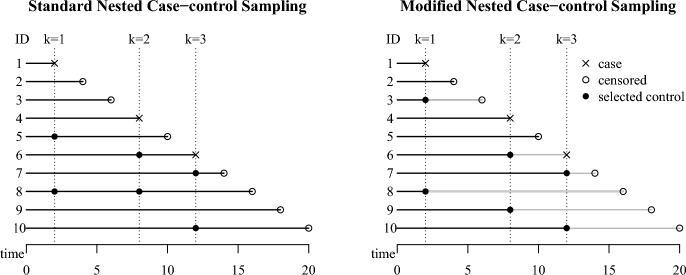
The standard and modified designs for a cohort with $N=10$ members, $D=3$ cases, and $m=2$ controls per case. The gray lines indicate exclusion from sampling pools

In Fig. 1, the left panel illustrates the standard NCC sampling scheme for a small cohort. Suppose that the cohort has ten units ($N=10$) whose indices (ID) are sorted by the time of either developing case or being censored whichever comes first, has three cases ($D=3$) for ID $= 1,4$, and 6, and selects two controls per case ($m=2$). The sampling pools for the standard NCC are then $\{2,3,4,5,6,7,8,9,10\}$ at $t_1$, $\{5,6,7,8,9,10\}$ at $t_2$, and $\{7,8,9,10\}$ at $t_3$. Here the subject with ID $= 8$ is sampled twice at $t_1$ and $t_2$, and the subject with ID $=6$ is sampled at $t_2$ and becomes a case at $t_3$. The right panel illustrates the modified NCC sampling scheme for the same cohort. The sampling pools for the modified NCC sampling are $\{2,3,4,5,6,7,8,9,10\}$ at $t_1$, $\{5,6,7,9,10\}$ at $t_2$, and $\{7,10\}$ at $t_3$. Each sampling pool is smaller than the corresponding sampling pools in the standard NCC design due to the exclusion of previously sampled controls.

### Conditional estimation on the matching structure

In the modified NCC sampling design, the partial likelihood estimator introduced in Sect. 2.3 remains the same in the use of risk sets. We denote the set of a case and sampled controls using the modified design as $\widetilde{\mathcal {R}}^*(t)$ for $t = t_1,\ldots ,t_D$. Replacing risk sets in response to the modified design, the partial likelihood estimators of $\varvec{\beta }$ and $\Lambda _0$ are computed as

5 $$\begin{aligned} \widehat{\varvec{\beta }}_{c*}&= \arg \max _{\varvec{\beta }} \prod _{i=1}^N\prod _{k=1}^D\Bigg \{\frac{\exp ({\varvec{\beta }}^\text {T}{\varvec{Z}}_i)}{\sum _{j\in \widetilde{\mathcal {R}}^*(t_k)} \exp ({\varvec{\beta }}^\text {T}{\varvec{Z}}_j)}\Bigg \}^{\text{dN}_i(t_k)}\text { and} \end{aligned}$$

6 $$\begin{aligned} \text{d}\widehat{\Lambda }_0^{c*}(t\mid \widehat{\varvec{\beta }}_{c*})&= \frac{\sum _{i=1}^N\text{dN}_i(t)}{\sum _{i\in \widetilde{\mathcal {R}}^*(t)} \{|\mathcal {R}(t)|/|\widetilde{\mathcal {R}}^*(t)|\}\exp (\widehat{\varvec{\beta }}_{c*}^\text {T}{\varvec{Z}}_i)}. \end{aligned}$$

For $t = t_1,\ldots ,t_D$, $\widetilde{\mathcal {R}}^*(t)$ are always disjoint for the modified design while $\widetilde{\mathcal {R}}(t)$ may have overlaps due to controls selected more than once in the standard design. As a consequence, the modified design increases the effective use of information in the risk set in the full cohort. Note that the values of $|\mathcal {R}(t)|/|\widetilde{\mathcal {R}}(t)|$ and $|\mathcal {R}(t)|/|\widetilde{\mathcal {R}}^*(t)|$ in the denominators of weighted Breslow estimators  (2) and (6) are same for any *t* because the risk set $\mathcal {R}(t)$ in the full cohort does not change by design and the same number of controls are selected at each case time $t = t_1,\ldots ,t_D$ from the risk set $\mathcal {R}(t)$ (that is, $|\widetilde{\mathcal {R}}(t)|=|\widetilde{\mathcal {R}}^*(t)|=m+1$).

Under regularity conditions, Prentice (1986) established the consistency and asymptotic normality of (5). These results take into account the correlation of risk sets between different failure times $t_k$’s caused by the exclusion of previously selected controls. The variance of (6) however remains the same with that of (2) as described in Sect. 2.3 because it is specified at each $t_k$ which integrates over all $t_k$’s.

### Estimation from a pooled subset: inverse probability weighting

Unlike the partial likelihood estimator, the inference with the IPW method must account for the change of design to the more complex modified NCC sampling beyond simple replacement of risk sets. The inverse probability weights $w_i$ introduced by Samuelsen (1997) is no longer valid because inclusion probabilities into the NCC data are different by designs. Individuals in the modified design are more likely to be sampled as controls since the modified scheme reduces the sampling pools by excluding previously sampled units. Mathematically, this means that $\pi _i\ge \pi _i^*$ where $\pi _i$ and $\pi _i^*$ are the inclusion probabilities of subject *i* as a control for the standard and modified design respectively. The equality ($\pi _i=\pi _i^*$) holds only when all of the previously sampled units are coincidentally excluded from the current and future sampling pools possibly due to censoring or other matching criteria, which would rarely happen in reality. As a result, if Samuelsen’s weights $w_i$ ($w_i=\delta _i + (1-\delta _i)/\pi _i$) are used for the sample obtained from the modified design, the resulting estimates of (3) and (4) will be biased. In other words, we have $\text{ E }(V_i/\pi _i) =1$ but $\text{ E }(V_i^*/\pi _i)\ge \text{ E }(V_i^*/\pi _i^*) =1$ where $V_i$ and $V_i^*$ denote binary variables that indicate whether subject *i* is ever sampled as a control $(=1)$ or not $(=0)$ for the standard and modified designs respectively.

In the following subsection, we derive the inclusion probabilities $\pi _i^*$ for the modified NCC design which makes (3) and (4) asymptotically unbiased. The proposed IPW estimators with $w_i^*$ ($w_i^*=\delta _i + (1-\delta _i)/\pi _i^*$) in place of $w_i$ in the pseudo-partial likelihood and weighted Breslow estimator are

7 $$\begin{aligned} \widehat{\varvec{\beta }}_{s*}&= \arg \max _{\varvec{\beta }}\prod _{i=1}^N\prod _{t>0}\Bigg \{\frac{\exp ({\varvec{\beta }}^\text {T}{\varvec{Z}}_i)}{\sum _{j\in \overline{\mathcal {R}}^*(t)} w_j^* \exp ({\varvec{\beta }}^\text {T} {\varvec{Z}}_j)}\Bigg \}^{\text{dN}_i(t)} \text { and}\end{aligned}$$

8 $$\begin{aligned} \text{d}\widehat{\Lambda }_0^{s*}(t\mid \widehat{\varvec{\beta }}_{s*})&= \frac{\sum _{i=1}^N \text{dN}_i(t)}{\sum _{i\in \overline{\mathcal {R}}^*(t)} w_i^* \exp {(\widehat{\varvec{\beta }}_{s*}^\text {T}{\varvec{Z}}_i})} \end{aligned}$$

where $\overline{\mathcal {R}}^*(t) = \big \{j \in \cup _{k=1}^D \widetilde{\mathcal {R}}^*(t_k)\mid Y_{jt_k}=1\big \}$ is a pooled subset of all cases and sampled controls using the modified design.

#### Sampling distribution and inclusion probabilities

Define $\mathcal {S}=\{1,\ldots , N\}$ as a full cohort and $s \subset \mathcal {S}$ as an arbitrary sample set from $\mathcal {S}$. We denote $\Omega$ as a set of all possible samples *s* in the modified NCC design given that at-risk individuals are known at every failure times, and $P(\cdot )$ as a sampling design distribution on $\Omega$ such that $\sum _{s\in \Omega }P(s)=1$. The inclusion probability $\pi _i^*$ for subject *i* is $\pi _i^*=\sum _{s\ni i}P(s)$ (Arnab 2017) where the sum is over all possible samples $s\in \Omega$ that contain subject *i*, and the joint (second order) inclusion probability $\pi _{ij}^*$ for subjects *i* and *j* with $i\ne j$ is $\pi _{ij}^*=\sum _{s\ni i\, \& \, j} P(s)$ where the sum is over all possible samples $s\in \Omega$ that contain both subjects *i* and *j*.

The number of all possible samples from $\mathcal {S}$ using the modified sampling scheme is $|\Omega |=\prod _{k=1}^D{r_k^*\atopwithdelims ()m}$ where $r_k^*=|\mathcal {R}_k\backslash \{\mathcal {Q}_k\cup \{k\}\}|=|\mathcal {R}_k\backslash \mathcal {Q}_k|-1$ denotes the size of sampling pool at $t_k$. As each NCC sample has equal chance on $\Omega$ (discrete uniform distribution) (Borgan et al. 1995), the sampling distribution of the modified NCC design is

9 $$\begin{aligned} P(s)=\frac{1}{|\Omega |}=\prod _{k=1}^D{r_k^*\atopwithdelims ()m}^{-1} \text { for } s\in \Omega . \end{aligned}$$

By definition, the inclusion probability for subject *i* is $\pi _i^*=\sum _{s\ni i}P(s)={|s\ni i|}/{|\Omega |}$ where $|s\ni i|$ is the number of samples *s* including subject *i*. The quantity $|s\ni i|$ is computed by subtracting the number of samples that do not include subject *i* from the total number of modified NCC samples:

$$\begin{aligned} |s\ni i| = |\Omega |-|s\not \ni i|=\prod _{k=1}^D{r_k^*\atopwithdelims ()m}-\prod _{k:\mathcal {R}_k \ni i}{r_k^*-1\atopwithdelims ()m}\prod _{k:\mathcal {R}_k \not \ni i}{r_k^*\atopwithdelims ()m}. \end{aligned}$$

As for the second term, we consider two situations. When subject *i* is at risk at $t_k$ ($\mathcal {R}_k\ni i$), the number of all possible control selections not including *i* at $t_k$ is $r_k^*-1\atopwithdelims ()m$ where the sampling pool excludes *i*. When subject *i* is not at risk at $t_k$ ($\mathcal {R}_k\not \ni i$), the number of control selections not containing *i* is $r_k^*\atopwithdelims ()m$ because the sampling pool does not include *i*. Combining these observations, we obtain the inclusion probability

10 $$\begin{aligned} \pi _i^* = 1-\prod _{k:\mathcal {R}_k \ni i}{r_k^*-1\atopwithdelims ()m}\bigg /{r_k^*\atopwithdelims ()m}=1-\prod _{k:\mathcal {R}_k \ni i}\frac{r_k^*-m}{r_k^*} = 1-\prod _{k:\mathcal {R}_k \ni i}\bigg (1-\frac{m}{r_k^*}\bigg ). \end{aligned}$$

This expression has the form in line with the Kaplan-Meier type estimator (Kaplan and Meier 1958). This (10) means the probability of being sampled at least once which is one minus the probability of never being sampled. To be specific, $1-m/r_k^*$ is the probability of not being sampled at $t_k$. Their product over all subjects *k* whose risk set $\mathcal {R}_k$ includes *i* is the probability of never being sampled at any $t_k$ when subject *i* is at risk.

The joint inclusion probabilities denoted by $\pi _{ij}^*$ are obtained for two exclusive circumstances: (a) *i* and *j* are sampled at different times or (b) *i* and *j* are sampled at the same time $t_k$ for any $k=1,\ldots , D$. For circumstance (a), $\pi _{ij}^* = \pi _i^*\pi _j^*$ because samplings at different times are conditionally independent given the structure of risk sets (i.e., the sampling pools). Note that (a) includes the circumstance when either *i* or *j* develops case event after being sampled as a control so that *i* and *j* also become independent because case incidence does not depend on control sampling. For circumstance (b), we expand as $\pi _{ij}^*=\sum _{s\ni i\, \& \, j} P(s)=\sum _{s\ni i} P(s)+\sum _{s\ni j} P(s) - \sum _{s\ni i\,\text {or}\,j} P(s)=\pi _i^*+\pi _j^*-\{1- \sum _{s\not \ni i\, \& \,j} P(s)\}$ for any $i\ne j$. We have $\sum _{s\not \ni i\, \& \,j} P(s) = {|s\not \ni i\, \& \, j|}/{|\Omega |}$ where, with abuse of notation, $|s\not \ni i\, \& \, j|$ denotes the number of samples $s\in \Omega$ that do not include both *i* and *j* sampled simultaneously, obtained by

$$\begin{aligned} |s\not \ni i\, \& \, j|=\prod _{\begin{array}{c} k:\mathcal {R}_k\ni i\\ \& \, \mathcal {R}_k\ni j \end{array}}{r_k^*-2 \atopwithdelims ()m}\prod _{\begin{array}{c} k:\mathcal {R}_k\not \ni i\\ ~~~\text {or}\, \mathcal {R}_k\not \ni j \end{array}}{r_k^* \atopwithdelims ()m}. \end{aligned}$$

When both *i* and *j* are at risk at $t_k$ ($\mathcal {R}_k\ni i$ and $\mathcal {R}_k\ni j$), the number of samples not including both *i* and *j* is $r_k^*-2 \atopwithdelims ()m$, which excludes subjects *i* and *j* from the sampling pool. When either *i* or *j* is not at risk at $t_k$ ($\mathcal {R}_k\not \ni i \text { or } \mathcal {R}_k\not \ni j$), there is no chance that both *i* and *j* are sampled simultaneously so that none needs to be excluded from the risk set to compute the number of samples not including both *i* and *j*. Thus, we have

11 $$\begin{aligned} \sum _{s\not \ni i\, \& \,j} P(s)&=\frac{|s\not \ni i\, \& \, j|}{|\Omega |} =\prod _{\begin{array}{c} k:\mathcal {R}_k\ni i\\ ~~~ \& \, \mathcal {R}_k\ni j \end{array}}{r_k^*-2\atopwithdelims ()m}\bigg /{r_k^*\atopwithdelims ()m} \nonumber \\&=\prod _{\begin{array}{c} k:\mathcal {R}_k\ni i\\ \& \, \mathcal {R}_k\ni j \end{array}}\bigg \{1-\frac{2m}{r_k^*}+\frac{m(m-1)}{r_k^*(r_k^*-1)}\bigg \}. \end{aligned}$$

Note that replacing $r_k=|\mathcal {R}_k\backslash \{k\}|$ by $r_k^*$ in the above derivation reduces to the inclusion probability $\pi _i$ and joint inclusion probability $\pi _{ij}$ in the standard NCC design in Sect. 2.4, which were given by Samuelsen (1997). Specifically, (10) and (11) are respectively equivalent to the terms $p_{0i}$ and $q_{0ij}$ of Samuelsen (1997) with $r_k$ in place of $r_k^*$.

#### Variance estimation based on influence functions

The variance of an estimator, say $\widehat{\varvec{\theta }}$, is approximated by the variance of the sum of their influence functions IF$_i({\varvec{\theta }})$ such that $N^{1/2}(\widehat{{\varvec{\theta }}} - {\varvec{\theta }}) = N^{-1/2}\sum _{i=1}^n\text {IF}_i({\varvec{\theta }}) + o_p(1)$ and $N\text {var}(\widehat{\varvec{\theta }}) \approx N^{-1}\text {var}\{\sum _{i=1}^N\text {IF}_i(\widehat{{\varvec{\theta }}})\}$ (Van der Vaart 2000, Ch.20). In this section, we derive the influence functions of $\widehat{\varvec{\beta }}$ and $\text{d}\widehat{\Lambda }_0(t)$ and furnish their variance formula for the modified NCC design.

For estimating $\varvec{\beta }$ in the modified design, we maximize the pseudo-partial likelihood with sampling weights $w_i^*$ as in (7). The estimating equation for $\varvec{\beta }$ is the unbiased weighted score function,

12 $$\begin{aligned} 0=\Psi _{1}(\varvec{\beta }) = \sum _{i\in s} w^*_i \sum _{t>0} \bigg \{ {\varvec{Z}}_i - \dfrac{ {S}^{(1)}(t;{\varvec{\beta }})}{ {S}^{(0)}(t; {\varvec{\beta }})}\bigg \}\text{dN}_i(t), \end{aligned}$$

where $s=\{i\mid \delta _i=1 \text { or }V_i^*=1\}$ denotes a set of all cases and controls and ${S}^{(r)}(t;\varvec{\beta }) =\sum _{j \in s} w^*_j \exp (\varvec{\beta }^\text {T} {\varvec{Z}}_j){{\varvec{Z}}_j}^{\otimes r}$ with $\varvec{z}^{\otimes 0} = 1$, $\varvec{z}^{\otimes 1} = \varvec{z}$ and $\varvec{z}^{\otimes 2} = \varvec{z}\varvec{z}^\text {T}$. Given $\widehat{\varvec{\beta }}$, the estimating equation for $\text{d}\Lambda _0(t)$ is

13 $$\begin{aligned} 0=\Psi _{2}\{\text{d}\Lambda _0(t)\mid {\widehat{\varvec{\beta }}}\}= \sum _{i \in s} w^*_i\big \{\text{dN}_i(t)-\text{d}\Lambda _0(t)\exp (\widehat{\varvec{\beta }}^\text {T} {\varvec{Z}}_i)\big \}, \end{aligned}$$

noticing that $\sum _{i \in s} w^*_i\text{dN}_i(t)= \sum _{i=1}^N \text{dN}_i(t)$ because $w_i^*=1$ when $\text{dN}_i(t)=1$. For simplicity, we suppress the parameters hereafter and use subscripts to indicate the corresponding estimating equations $\Psi _1$ and $\Psi _2$ for $\varvec{\beta }$ and $\text{d}\Lambda _0(t)$, respectively.

By applying the influence function operator (Pfeiffer and Gail 2017) to (12) and (13), we obtain

14 $$\begin{aligned} 0&=\text {IF}_i(\Psi _1)+\Psi _{11} \text {IF}_i(\widehat{\varvec{\beta }});\nonumber \\ 0&=\text {IF}_i(\Psi _2) + \Psi _{22}\text {IF}_i\{\text{d}\widehat{\Lambda }_0(t)\}+ \Psi _{21}\text {IF}_i(\widehat{\varvec{\beta }}), \end{aligned}$$

where $\Psi _{11}= -\sum _{i \in s}w^*_i \sum _{t>0}[{S^{(2)}(t;{\varvec{\beta }})}/{{S}^{(0)}(t;{\varvec{\beta }})}-\{{S^{(1)}(t;{\varvec{\beta }})}/{S^{(0)}(t;{\varvec{\beta }})}\}^2]\,\text{dM}_i(t)$ with a martingale residual at *t* denoted by $\text{dM}_i(t) = \text{dN}_i(t)-V_i^*w^*_i\exp ({\varvec{\beta }^\text {T} {\varvec{Z}}_i})\sum _{j=1}^N\text{dN}_j(t)/{{S}^{(0)}(t; \varvec{\beta })}$, $\Psi _{22}= - {S}^{(0)}(t;{\varvec{\beta }})$, and $\Psi _{21}= -\text{d}{\Lambda }_0(t){S}^{(1)}(t;{\varvec{\beta }})$. The leading terms in (14) are obtained from (12) and (13) as, for $i \in s$,

15 $$\begin{aligned} \text {IF}_i(\Psi _1)&= w^*_i \sum _{t>0}\bigg \{ {\varvec{Z}}_i - \dfrac{ {S}^{(1)}(t;{\varvec{\beta }})}{ {S}^{(0)}(t;{\varvec{\beta }})}\bigg \}\text{dM}_i(t); \nonumber \\ \text {IF}_i(\Psi _2)&= w^*_i\big \{\text{dN}_i(t)-\text{d}\Lambda _{0}(t)\exp ({\widehat{\varvec{\beta }}^\text {T} {\varvec{Z}}_i})\big \}. \end{aligned}$$

The influence functions of estimates are then obtained by solving (14) sequentially, using the quantities (15), as

16 $$\begin{aligned} \text {IF}_i(\widehat{\varvec{\beta }})&= - \Psi _{11}^{-1}\big \{\text {IF}_i(\Psi _1)\big \}; \nonumber \\ \text {IF}_i\{\text{d}\widehat{\Lambda }_0(t)\}&=-\Psi _{22}^{-1}\big \{\text {IF}_i(\Psi _2) +\Psi _{21}\text {IF}_i(\widehat{\varvec{\beta }})\big \}. \end{aligned}$$

These derivations are similar to the computation of influences for a general two-stage *M*-estimator (Zhelonkin et al. 2012). The influence function of cumulative baseline hazard in arbitrary time interval $(\tau _0, \tau _1]$ for risk projection can be obtained as $\text {IF}_i\{\textstyle \int _{\tau _0}^{\tau _1}\text{d}\widehat{\Lambda }_0(t)\} = \textstyle \sum _{t\le \tau _1}\text {IF}_i\{\text{d}\widehat{\Lambda }_0(t)\}-\sum _{t\le \tau _0}\text {IF}_i\{\text{d}\widehat{\Lambda }_0(t)\}$.

For estimating the variance of the influence functions, we decompose the variance by conditioning on survival and covariate information available in the full cohort, denoted by $\mathcal {C}$, and then uncondition by viewing $\mathcal {C}$ as a sample from a superpopulation. The conditional part corresponds to the finite population variance coming from control selection, and the full (unconditional) variance corresponds to the superpopulation component. As the derivation in the following applies to influence functions of any estimator in the modified NCC design, we denote $\text {IF}_i\in \{\text {IF}_i(\widehat{\varvec{\beta }}), \text {IF}_i(\widehat{\Lambda }_0(t))\}$ for simplicity. Given $\mathcal {C}$, the components of $\text {IF}_{i}$, $\pi _i^*$, and $\pi _{ij}^*$ are fixed, but only $V_i^*$ is random, depending on the sampling. We have $\text {E}(V_i^*\mid \mathcal {C}) = \pi _i^*$ and $\text {E}(V_i^*w_i^*\mid \mathcal {C}) = 1$. Also, $\sigma _{ij}^*= \text {var}(V_i^*, V_j^*\mid \mathcal {C})$ is $\pi _i^*(1-\pi _i^*)$ for $i=j$ and $\pi _{ij}^*-\pi _i^*\pi _j^*$ for $i\ne j$. Therefore, the variance based on influence functions is

17 $$\begin{aligned} \text {var}\Bigg \{\sum _{i=1}^N\text {IF}_i\Bigg \}&= {{\text {var}\Bigg \{\sum _{i=1}^N \text {IF}_{i}\Bigg \}}} + {\text {E}\Bigg \{\sum _{i,j=1}^N w_i^*w_j^*\text {IF}_{i}\text {IF}_{j}^\text {T}\sigma _{ij}^*\Bigg \}}. \end{aligned}$$

The first term is the variance for a cohort with complete data from an infinite superpopulation. The second term is the variance due to the modified NCC sampling from the full cohort. Finally, we estimate the two terms separately using the available nested case–control data as

18 $$\begin{aligned} \widehat{\text {var}}\Bigg \{\sum _{i=1}^N\text {IF}_i \Bigg \}&= \frac{N}{N-1} \sum _{i\in s}\Bigg \{w_i^*\text {IF}_{i}\text {IF}_{i}^{T}\Bigg \} + \sum _{i,j\in s}w^*_{ij}w_i^*w_j^* \text {IF}_{i}\text {IF}_{j}^\text {T}\sigma _{ij} \end{aligned}$$

where $$w_{ij}^* = (1/{\pi _i^*})I_{(i=j)}+(1/{\pi _{ij}^*})I_{(i\ne j)}$$.

We want to note that Samuelsen (1997) derived the variance estimation for $\widehat{\varvec{\beta }}_s$ using pseudo-score functions and pseudo-information matrix, which can also be established with $\pi ^*$ of the modified design. We however used influence functions, despite asymptotically equivalence, because it naturally extends to the variance estimation for $\text{d}\widehat{\Lambda }^s_0(t\mid \widehat{\varvec{\beta }}_s)$, which are subsequent and nonparametric estimates given $\widehat{\varvec{\beta }}_s$. Such extension is general for any subsequent estimates given $\widehat{\varvec{\beta }}_s$ and $\text{d}\widehat{\Lambda }^s_0(t\mid \widehat{\varvec{\beta }}_s)$ so that the variances of survival probability or absolute risk can be straightforwardly estimated using influence functions as well.

## Simulation

### Setup

We first generated two risk factors ${Z}_1$ and ${Z}_2$ from the standard normal distribution with $\text {corr}({Z}_1,{Z}_2)=$ 0.25. To generate survival time *T* (time to death) from the Cox model, we used the hazard function $\lambda (t;{\varvec{Z}}) = \lambda _0(t)\exp (\beta _1{ Z}_1+\beta _2{Z}_2)$ where $\lambda _0(t) = -\log (0.95)/10$ is constant with a 5% case incidence rate at $t=10$ (the end of the study) at a referent level ${\varvec{Z}}=(0,0)^\text {T}$. We set $\beta _1=0.5$ and $\beta _2= 0.9$ for moderate levels of log-relative hazards. The censoring time is $\min (C,10)$ where $C \sim \text {Exp}(\alpha )$ (i.e., a constant risk of censoring over time). We considered four levels of right censoring by the choice of $\alpha = -\log \{(1-p_c)/10\}$ with $p_c=0.2, 0.4, 0.6$ and 0.8 for 20%, 40%, 60% and 80% death at $t=10$, respectively. The censored survival time is $X=\min (T, C, 10)$ and the case indicator is ${\delta }=I\{T\le \min (C,10)\}$. We considered the time-on-study time scale in the simulation study (we will consider the age scale in Sect. 5).

For a randomly generated full cohort of $N=5000$, we collected two types of the NCC data by the standard and modified NCC designs respectively with different numbers of controls, $m=1,2,3,4$, and 5, at each case time. For a fair comparison, we implemented control selections as follows. At each time that a case develops, $(t_k; k=1,\ldots , D)$, we first sampled *m* distinctive controls, $\{j_1(t_k),\ldots , j_m(t_k)\}$, from a set of at-risk non-cases (the sampling pool of the standard design, $\mathcal {R}_k\backslash \{k\}$). If, without loss of generality, $\{j_1(t_k),\ldots , j_{m'}(t_k)\}$ with $m'<m$ had already been selected before $t_k$, we dropped those duplicated units and newly sampled $m'$ distinctive controls, $\{j^\prime _1(t_k), \ldots , j^\prime _{m'}(t_k)\}$, from a set of at-risk non-cases who are not in $\{j_{m'+1}(t_k), \ldots , j_{m}(t_k)\}$ and had not been selected before $t_k$ (the sampling pool of the modified design, $\mathcal {R}_k\backslash \{\mathcal {Q}_k\cup \{k\}\}$). As a result, the NCC sample from the standard design is $s_\text {std} = \cup _{k=1}^D\{k, j_1(t_k),\ldots , j_m(t_k)\}$ and that from the modified design is $s_\text {mod} = \uplus _{k=1}^D\{k, j^\prime _1(t_k), \ldots , j^\prime _{m'}(t_k)$, $j_{m'+1}(t_k), \ldots , j_{m}(t_k)\}$ where $\uplus$ denotes a disjoint set union. This strategy makes $s_\text {std}\subseteq s_\text {mod}$ so that the additional impact of the modified design compared to the standard design can be observed.

To compare the performance of different designs, we computed the estimators of $\varvec{\beta }= (\beta _1,\beta _2)$ and $\text{d}\Lambda _0(t)$ by the partial likelihood estimation and IPW method in the standard design discussed in Sects. 2.3–2.4 and in the modified design trated in Sects. 3.2–3.3. We ran this simulation $B=1000$ times.

### Results summary

#### Bias of estimators from standard and modified nested case–control designs

**Table 1:** Simulation results for design bias (DB) of estimates of log-relative hazards and cumulative baseline hazards obtained from the standard (std) and modified (mod) nested case–control sampling design

	$\widehat{\beta }_1^c$		$\widehat{\beta }_1^s$		$\widehat{\beta }_2^c$		$\widehat{\beta }_2^s$		$\widehat{\Lambda }_0^c(10)$		$\widehat{\Lambda }_0^s(10)$	
*m*	std	mod	std	mod	std	mod	std	mod	std	mod	std	mod
C20%												
1	0.3585	0.4821	0.5404	0.4494	1.0227	1.0110	1.4927	1.4786	0.0138	0.0163	0.0227	0.0221
2	0.3951	0.2367	0.4580	0.3588	0.5566	0.5045	0.7072	0.6204	0.0008	0.0017	0.0123	0.0104
3	0.2152	0.2469	0.2220	0.2874	0.3636	0.2286	0.4144	0.2361	0.0003	0.0001	0.0075	0.0062
4	0.1863	0.2212	0.1709	0.1732	0.1700	0.0735	0.3432	0.1416	0.0013	0.0030	0.0094	0.0053
5	0.1105	0.1976	0.0951	0.1114	0.1171	0.1109	0.1427	0.0543	0.0002	0.0018	0.0058	0.0014
C40%												
1	1.0190	0.9270	1.3476	1.1695	1.1401	0.9706	1.6696	1.5010	0.0187	0.0143	0.0393	0.0406
2	0.6387	0.5565	0.6883	0.6205	0.9968	0.7303	0.7439	0.5920	0.0033	0.0013	0.0179	0.0199
3	0.5163	0.3869	0.4359	0.3837	0.7690	0.6302	0.4299	0.3382	0.0007	0.0019	0.0091	0.0105
4	0.3240	0.1015	0.3589	0.2756	0.5178	0.4071	0.3233	0.1861	0.0014	0.0008	0.0115	0.0086
5	0.3100	0.1119	0.3291	0.2870	0.4429	0.3756	0.2964	0.1588	0.0019	0.0030	0.0103	0.0079
C60%												
1	0.7103	0.8692	0.7677	0.9477	1.6390	1.7785	2.3342	2.3406	0.0181	0.0220	0.0449	0.0470
2	0.5116	0.4352	0.2826	0.3380	0.8779	1.0921	1.4332	1.5449	0.0031	0.0027	0.0291	0.0305
3	0.3466	0.2070	0.3178	0.2030	0.5235	0.7160	0.8852	0.8587	0.0055	0.0041	0.0189	0.0175
4	0.1255	0.0564	0.1118	0.0606	0.4114	0.4649	0.6452	0.6090	0.0079	0.0075	0.0137	0.0129
5	0.1098	0.0531	0.1172	0.0699	0.3242	0.3723	0.4806	0.4531	0.0056	0.0074	0.0122	0.0121
C80%												
1	0.6759	0.5438	1.4574	1.4451	2.8332	2.5416	3.6358	3.1564	0.0191	0.0103	0.0641	0.0660
2	0.4304	0.3065	0.8644	0.9180	1.4605	1.1056	1.8851	1.5723	0.0079	0.0127	0.0386	0.0418
3	0.1600	0.2818	0.5664	0.7101	1.1590	0.8840	1.3444	1.0715	0.0084	0.0096	0.0289	0.0302
4	0.1966	0.3231	0.5380	0.6854	0.8494	0.6732	0.9507	0.7100	0.0101	0.0116	0.0247	0.0257
5	0.2847	0.2352	0.5689	0.6189	0.6404	0.5559	0.7058	0.5002	0.0069	0.0077	0.0209	0.0179

The values of DB are multiplied by 100 here to save space. For reference, the empirical means of full cohort estimates were $\widehat{\beta }_1=0.5$, $\widehat{\beta }_2=0.9$, and $\widehat{\Lambda }_0(10)=0.051$

*m* = number of matched control(s) per case

C*xx*% = $xx\%$ of cohort members are censored before or at the end of study

$\widehat{\beta }^c$ = log-relative risk estimated by partial likelihood estimation;

$\widehat{\beta }^s$ = log-relative risk estimated by pseudo-partial likelihood maximization;

$\widehat{\Lambda }_0^c(10)$ = cumulative baseline hazard at $t=10$ estimated by Langholz–Borgan weighting

$\widehat{\Lambda }_0^s(10)$ = cumulative baseline hazard at $t=10$ estimated by inverse probability weighting

Denote $\widehat{\varvec{\theta }}^{(b)}$ as the estimates obtained at *b*th run ($b=1,\ldots ,B$) for any $\varvec{\theta }\in \{\varvec{\beta }, \Lambda _0(\tau )\}$ with $\tau = 10$. To assess the bias of NCC estimates, $\widehat{\varvec{\theta }}_\text {ncc}$, of each design (the standard or modified design) compared to the full cohort estimates, $\widehat{\varvec{\theta }}_\text {full}$, we computed the absolute design bias (DB),

$$\begin{aligned} \text {DB}(\widehat{\varvec{\theta }}_\text {ncc}) = |\widehat{\text {E}}(\widehat{\varvec{\theta }}_\text {ncc})-\widehat{\text {E}}(\widehat{\varvec{\theta }}_\text {full})|, \end{aligned}$$

where $\widehat{\text {E}}(\widehat{\varvec{\theta }})=\sum _{b=1}^B\widehat{\varvec{\theta }}^{(b)}/B$ denotes the empirical mean. Table 1 shows DB’s for $\varvec{\beta }$ and $\Lambda _0$ for all scenarios where the numbers are multiplied by 100 to save space. For reference, $\widehat{\text {E}}(\widehat{\beta }_\text {1, full}) = 0.5$; $\widehat{\text {E}}(\widehat{\beta }_\text {2, full}) = 0.9$; and $\widehat{\text {E}}(\widehat{\Lambda }_\text {0, full}) = 0.051$ at any level of censoring.

In summary, regardless of the estimation methods, the DB’s of the standard design were similar with those of the modified design, and all DB’s were close to zero. This suggests that the both standard and modified samplings lead to the design-unbiased estimates ($\text {E}(\widehat{\varvec{\theta }}_\text {ncc,std})=\text {E}(\widehat{\varvec{\theta }}_\text {ncc,mod})=\text {E}(\widehat{\varvec{\theta }}_\text {full})=\varvec{\theta }$ where $\widehat{\varvec{\theta }}_\text {ncc/std}$ and $\widehat{\varvec{\theta }}_\text {ncc/mod}$ denotes the estimates from the standard and modified design respectively for true parameters $\varvec{\theta }$).

We want to note that the DB decreases as the number of matched controls (*m*) increases. This suggests that the bias reduces with the bigger subsample and therefore the modified design is likely to have a smaller bias than the standard design because $|s_\text {std}| \le |s_\text {mod}|$, even though the numerical difference is very small in our simulation study. Also, the DB decreases as the proportion of censored units $(Cxx\%)$ decreases, indicating that the bias reduces with the bigger sampling pool (the less censored, the more candidates in sampling pools).

#### Efficiency of estimators from standard and modified nested case–control designs

To assess the variance of NCC estimates compared to the full cohort estimates, we computed design variance (DV) of $\widehat{\varvec{\theta }}_\text {ncc}$, defined as

$$\begin{aligned} {\text {DV}}(\widehat{\varvec{\theta }}_\text {ncc})=\widehat{\text {var}}(\widehat{\varvec{\theta }}_\text {ncc})- \widehat{\text {var}}(\widehat{\varvec{\theta }}_\text {full}), \end{aligned}$$

where $\widehat{\text {var}}(\widehat{\varvec{\theta }})=\sum _{b=1}^B\{\hat{\varvec{\theta }}^{(b)}-\widehat{\text {E}}(\widehat{\varvec{\theta }})\}^2/(B-1)$ denotes the empirical variance. Note that the variance of NCC estimate is decomposed into the variance of full cohorts sampled from superpopulation (population variance) and the variance from sampling controls from a fixed full cohort (design variance). Analytically, this can be viewed as the variance decomposition formula by conditioning on a full cohort ($\mathcal C$); that is, ${\text {var}}(\widehat{\varvec{\theta }}_ {\rm ncc}) = \text{var}\{\text{E}(\widehat{\varvec{\theta}}_{\rm ncc}\mid \mathcal{C})\} + {\text{E}\{\text{var}}(\widehat{\varvec{\theta}}_{\rm ncc}\mid \mathcal{C})\}$ where the first term corresponds to the population variance ${\text{var}}(\widehat{\varvec{\theta }}_{\rm full})$, and the second term corresponds to the design variance ${{\text{E}}\{\text{DV}}(\widehat{\varvec{\theta}}_{\rm ncc})\}$. The population variance cannot be reduced once the full cohort is given; however, the design variance can be reduced by modifying the sampling scheme of design, which we want to investigate in this study. We therefore computed the ratio of design variances (R) of the two NCC designs

$$\begin{aligned} {\text {R}}={\text {DV}}(\widehat{\varvec{\theta }}_\text {ncc,std})/{\text {DV}}(\widehat{\varvec{\theta }}_\text {ncc,mod}), \end{aligned}$$

so that $\text {R}>1$ suggests the better performance of the modified design compared to the standard design for estimating $\varvec{\theta }$.

**Fig. 2:**
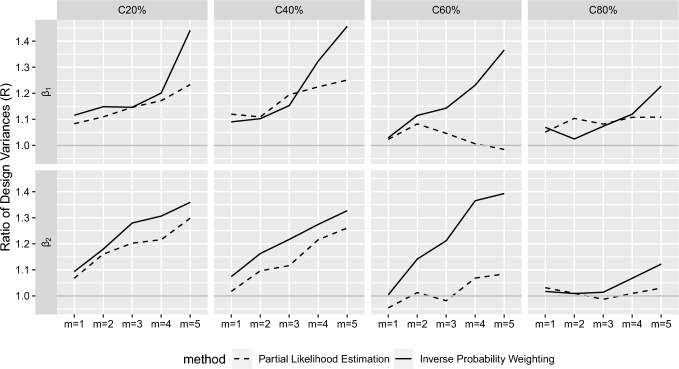
Ratio of design variances (*R*) of $\beta$-estimates from the modified NCC design relative to the standard NCC design; $Cxx\% = xx\%$ of cohort members are censored before or at the end of study; $m =$ the number of matched control(s) per case; the ratio greater than 1 (gray horizontal lines), $R>1$, indicates the smaller variance (higher efficiency) for the modified design.

**Fig. 3:**
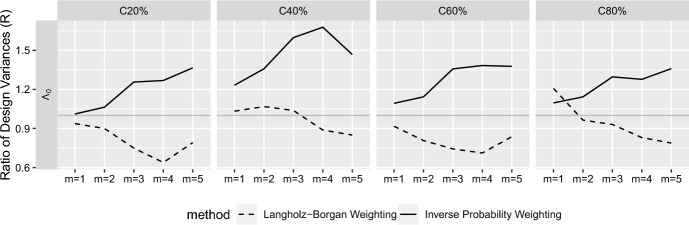
Ratio of design variances (*R*) of $\Lambda$-estimates from the modified NCC design relative to the standard NCC design; $Cxx\% = xx\%$ of cohort members are censored before or at the end of study; $m =$ the number of matched control(s) per case; the ratio greater than 1 (gray horizontal lines), $R>1$, indicates the smaller variance (higher efficiency) for the modified design

**Table 2:** Simulation results for the square root of design variance ($\sqrt{\text {DV}}$) of estimates of log-relative hazards and cumulative baseline hazards obtained from the standard (std) and modified (mod) nested case–control sampling design

	$\widehat{\beta }_{1}^{c}$		$\widehat{\beta }_1^s$		$\widehat{\beta }_2^c$		$\widehat{\beta }_2^s$		$\widehat{\Lambda }_0^c(10)$		$\widehat{\Lambda }_0^s(10)$	
*m*	std	mod	std	mod	std	mod	std	mod	std	mod	std	mod
C20%												
1	0.0924	0.0888	0.0846	0.0801	0.0994	0.0962	0.0948	0.0906	0.0017	0.0018	0.0024	0.0024
2	0.0662	0.0629	0.0570	0.0532	0.0725	0.0673	0.0649	0.0598	0.0014	0.0015	0.0016	0.0016
3	0.0559	0.0522	0.0467	0.0436	0.0622	0.0568	0.0526	0.0465	0.0012	0.0013	0.0014	0.0012
4	0.0494	0.0456	0.0389	0.0355	0.0530	0.0480	0.0430	0.0376	0.0010	0.0013	0.0011	0.0010
5	0.0443	0.0399	0.0344	0.0287	0.0497	0.0436	0.0379	0.0325	0.0011	0.0012	0.0010	0.0009
C40%												
1	0.0965	0.0911	0.0892	0.0854	0.1055	0.1046	0.1010	0.0975	0.0021	0.0021	0.0028	0.0025
2	0.0708	0.0672	0.0640	0.0609	0.0787	0.0752	0.0697	0.0646	0.0016	0.0016	0.0021	0.0018
3	0.0615	0.0563	0.0533	0.0496	0.0630	0.0597	0.0567	0.0514	0.0015	0.0014	0.0017	0.0013
4	0.0541	0.0489	0.0449	0.0390	0.0568	0.0515	0.0487	0.0431	0.0014	0.0015	0.0015	0.0012
5	0.0497	0.0444	0.0401	0.0332	0.0526	0.0468	0.0427	0.0371	0.0013	0.0014	0.0013	0.0010
C60%												
1	0.1023	0.1011	0.1005	0.0991	0.1148	0.1176	0.1014	0.1012	0.0023	0.0024	0.0029	0.0028
2	0.0747	0.0718	0.0719	0.0681	0.0826	0.0820	0.0744	0.0696	0.0017	0.0019	0.0020	0.0019
3	0.0621	0.0607	0.0565	0.0528	0.0670	0.0676	0.0593	0.0539	0.0015	0.0017	0.0016	0.0014
4	0.0536	0.0534	0.0494	0.0445	0.0588	0.0569	0.0525	0.0450	0.0014	0.0017	0.0015	0.0012
5	0.0473	0.0477	0.0424	0.0362	0.0525	0.0504	0.0450	0.0381	0.0015	0.0016	0.0014	0.0012
C80%												
1	0.1223	0.1193	0.1245	0.1204	0.1373	0.1352	0.1362	0.1351	0.0035	0.0031	0.0040	0.0039
2	0.0872	0.0830	0.0816	0.0807	0.0983	0.0978	0.0969	0.0964	0.0024	0.0025	0.0030	0.0028
3	0.0706	0.0679	0.0646	0.0623	0.0783	0.0788	0.0730	0.0725	0.0020	0.0021	0.0023	0.0021
4	0.0629	0.0598	0.0540	0.0510	0.0688	0.0685	0.0646	0.0625	0.0019	0.0021	0.0022	0.0020
5	0.0558	0.0530	0.0476	0.0430	0.0606	0.0597	0.0554	0.0523	0.0018	0.0021	0.0021	0.0018

*m* = number of matched control(s) per case;

C*xx*% = $xx\%$ of cohort members are censored before or at the end of study;

$\widehat{\beta }^c$ = log-relative risk estimated by partial likelihood estimation;

$\widehat{\beta }^s$ = log-relative risk estimated by pseudo-partial likelihood maximization;

$\widehat{\Lambda }_0^c(10)$ = cumulative baseline hazard at $t=10$ estimated by Langholz–Borgan weighting;

$\widehat{\Lambda }_0^s(10)$ = cumulative baseline hazard at $t=10$ estimated by inverse probability weighting

To summarize, as $\text{ R }>1$ for the most scenarios, the modified NCC design outperformed the standard design for estimating log-relative hazards $\varvec{\beta }$ (Fig. 2). The values R increased with lower censoring and/or larger *m*. This is because in the standard design the previously sampled controls are more likely to stay in the future risk sets with the lower level of censoring and, hence, more likely to be sampled again with larger *m*. However, the modified design eliminates all the previously sampled controls from the future risk sets and therefore avoids duplicates. This efficiency gain of the modified design was highlighted when using pseudo-partial likelihood estimation method, which unifies all cases and controls into the analysis and directly accounts for the modified sampling pools into the weights ($w^*$ in Sect. 3.3.1). See Table 2 for the numerical results. For reference, $\sqrt{\widehat{\text {var}}}(\widehat{\beta }_\text {1, full})$ were 0.0533, 0.0571, 0.0625, and 0.0716; $\sqrt{\widehat{\text {var}}}(\widehat{\beta }_\text {2, full})$ were 0.0544, 0.0580, 0.0654, and 0.0740 for the censoring level, 20%, 40%, 60% and 80%, respectively.

In Fig. 3, the higher efficiency of the modified design was also observed for $\widehat{\Lambda }_0$ with the IPW estimator, but not with the Langholz–Borgan-weighted estimator. This is because the inverse probability weights (*w* and $w^*$) account for the size of sampling pools, which depends on the type of NCC sampling design ($r_k$ and $r_k^*$, respectively). However, the Langholz–Borgan weights $|\mathcal {R}(t)|/(m+1)$ in (2) only account for the size of risk sets at case times, which are the same for the both standard and modified designs. See Table 2 for the numerical results. For reference, $\sqrt{\widehat{\text {var}}}(\widehat{\Lambda }_\text {0, full})$ were 0.0037, 0.0040, 0.0046, and 0.0054, for the censoring level, 20%, 40%, 60% and 80%, respectively.

#### Accuracy of variance estimation and coverage of confidence intervals

**Table 3:** Simulation results for variance and coverage rate of the estimates of log-relative hazards and cumulative baseline hazards obtained from the standard (std) and modified (mod) nested case–control sampling design. ($p_c=0.2$, $B=200$)

		$\widehat{\beta }_1^c$		$\widehat{\beta }_1^s$		$\widehat{\beta }_2^c$		$\widehat{\beta }_2^s$		$\widehat{\Lambda }_0^c(10)$		$\widehat{\Lambda }_0^s(10)$	
*m*		std	mod	std	mod	std	mod	std	mod	std	mod	std	mod
1	SD	0.1066	0.1089	0.0964	0.0957	0.1142	0.1100	0.1059	0.1004	0.0044	0.0043	0.0046	0.0046
	SE	0.1015	0.1041	0.0908	0.0899	0.1128	0.1130	0.0947	0.0936	0.0043	0.0045	0.0043	0.0043
	CR	0.93	0.95	0.93	0.92	0.97	0.96	0.90	0.92	0.94	0.95	0.92	0.91
2	SD	0.0846	0.0852	0.0768	0.0773	0.0877	0.0873	0.0800	0.0802	0.0042	0.0043	0.0043	0.0044
	SE	0.0822	0.0846	0.0733	0.0717	0.0895	0.0935	0.0764	0.0745	0.0041	0.0045	0.0041	0.0040
	CR	0.93	0.96	0.94	0.93	0.96	0.96	0.93	0.92	0.94	0.95	0.94	0.92
3	SD	0.0765	0.0746	0.0703	0.0686	0.0768	0.0768	0.0727	0.0735	0.0042	0.0042	0.0042	0.0043
	SE	0.0741	0.0771	0.0661	0.0641	0.0803	0.0825	0.0686	0.0664	0.0040	0.0048	0.0040	0.0039
	CR	0.94	0.95	0.92	0.93	0.97	0.97	0.93	0.94	0.93	0.95	0.93	0.92
4	SD	0.0707	0.0685	0.0653	0.0628	0.0723	0.0727	0.0682	0.0695	0.0042	0.0041	0.0041	0.0041
	SE	0.0698	0.0732	0.0623	0.0601	0.0753	0.0803	0.0646	0.0623	0.0040	0.0050	0.0039	0.0039
	CR	0.95	0.97	0.93	0.95	0.96	0.98	0.95	0.93	0.93	0.97	0.95	0.92
5	SD	0.0670	0.0648	0.0611	0.0596	0.0703	0.0697	0.0671	0.0665	0.0041	0.0041	0.0040	0.0041
	SE	0.0670	0.0706	0.0599	0.0577	0.0721	0.0751	0.0621	0.0597	0.0040	0.0054	0.0039	0.0038
	CR	0.95	0.96	0.94	0.94	0.95	0.97	0.93	0.94	0.93	0.98	0.93	0.92

*m* = number of matched control(s) per case;

SD = standard deviation;

SE = mean of standard errors;

CR = coverage rate of 95% confidence intervals;

$\widehat{\beta }^c$ = log-relative risk estimated by partial likelihood estimation;

$\widehat{\beta }^s$ = log-relative risk estimated by pseudo-partial likelihood maximization;

$\widehat{\Lambda }_0^c(10)$ = cumulative baseline hazard at $t=10$ estimated by Langholz–Borgan weighting;

$\widehat{\Lambda }_0^s(10)$ = cumulative baseline hazard at $t=10$ estimated by inverse probability weighting

Table 3 summarizes the variance estimates and coverages of 95% confidence intervals (CIs) based on the 20% censoring scenario with $B=200$ runs. The average estimated variances for all estimators were near their empirical variances (i.e., SE $\approx$ SD). All the coverages of 95% CIs were nearly at the nominal value, 0.95. The 95% CI of coverages based on 200 simulations is $0.95 \pm 0.015$, which includes almost all the coverages shown.

## Data example: mortality risk in NIH-AARP cohort study

To illustrate the sampling schemes of the NCC design, we studied mortality risk using data from the NIH-AARP Diet and Health Cohort Study (Schatzkin et al. 2001) that recruited members aged 50–71 in 1995–1996 with follow up through 2011. We confined analysis to $N=27886$ women, who had complete information on age, exit status ($1=$ dead or $0=$ censored due to lost-to-follow up for any reason, alive by December 31, 2011 or age 85-year-old, whichever came first), waist circumference, and smoking status. Among them, $D=4604$ women died during the follow-up period.

We categorized waist measurements (in centimeters) such that WAIST:low (or WAIST:high) was 1 if a woman had waist measurement less than the first quartile, 76.2 cm (or greater than the third quartile, 92.1 cm) and 0 otherwise. The two middle quartiles were used as the reference group. The SMOKE:current was 1 for a current smoker, and 0 otherwise. Non-smokers were the referent group. Using age as the time scale, we analyzed the effect of waist circumference and smoking behavior on mortality risk; that is, $\lambda (t) = \lambda _0(t)\exp (\beta _1\text {WAIST:low}+\beta _2\text {WAIST:high}+\beta _3\text {SMOKE:current})$ with age *t*.

We sampled data from the cohort with the standard and modified design with $m=1,2,3$ control(s) per case. The waist measurements are collected for the sampled individuals at the times of death cases during the follow-up period from 1996 to 2011.

**Table 4:** Summary of AARP data example study results; point estimates (standard error estimates (in parentheses) obtained from the standard (std) and modified (mod) nested case–control sampling design

	$\beta _\text {1,WAIST:low}$		$\beta _\text {2,WAIST:high}$		$\beta _\text {3,SMOKE:current}$		$\int _{60}^{70}\lambda _0(t)\,\text{d}t$	
Full Cohort								
	$-$0.0006		0.3392		1.1382		0.0418	
	(0.0375)		(0.0346)		(0.0341)		(0.0015)	
Nested Case–control: Partial Likelihood Estimation								
*m*	std	mod	std	mod	std	mod	std	mod
1	0.0201	$-$0.0064	0.3659	0.4134	1.0781	1.1630	0.0416	0.0407
	(0.0551)	(0.0545)	(0.0524)	(0.0531)	(0.0611)	(0.0629)	(0.0024)	(0.0023)
2	$-$0.0068	$-$0.0077	0.3525	0.4061	1.1127	1.1825	0.0416	0.0406
	(0.0448)	(0.0452)	(0.0426)	(0.0436)	(0.0475)	(0.0496)	(0.0022)	(0.0022)
3	0.0022	$-$0.0061	0.3572	0.3927	1.1366	1.2116	0.0414	0.0407
	(0.0401)	(0.0414)	(0.0383)	(0.0395)	(0.0417)	(0.0443)	(0.0022)	(0.0021)
Nested Case–control: Inverse Probability Weighting Estimation								
*m*	std	mod	std	mod	std	mod	std	mod
1	0.0025	$-$0.0146	0.3254	0.3477	1.0882	1.1194	0.0423	0.0399
	(0.0520)	(0.0496)	(0.0490)	(0.0469)	(0.0531)	(0.0504)	(0.0018)	(0.0017)
2	$-$0.0328	$-$0.0160	0.3117	0.3515	1.0935	1.1137	0.0426	0.0406
	(0.0446)	(0.0422)	(0.0417)	(0.0393)	(0.0437)	(0.0407)	(0.0017)	(0.0016)
3	$-$0.0289	0.0012	0.3220	0.3487	1.1238	1.1238	0.0423	0.0418
	(0.0419)	(0.0394)	(0.0390)	(0.0365)	(0.0402)	(0.0368)	(0.0016)	(0.0016)

Table 4 summarizes the results of the data example study showing point estimates with standard errors for the log-relative hazards, $\beta$’s, and the 10-year cumulative baseline hazard from age 60 to 70, $\int _{60}^{70} \lambda _0(t)\,\text{d}t$. The partial likelihood estimation and pseudo-partial likelihood maximization were used for estimating $\beta$, and the Langholz–Borgan weights and the inverse probability weights were used for estimated $\lambda _0(t)$ for each of the NCC designs.

To summarize, all the estimates were closer to the estimates from the full cohort (gold standard) as *m* increases for the both designs. This result is consistent with the simulation study in which the design bias reduces as *m* increases. For any estimation method, the standard and modified designs were similar in the point estimates especially for the variables with significant effect on the mortality suggesting that the both designs led to the unbiased estimates. When using partial likelihood estimation and Langholz–Borgan weighted Breslow estimator, the standard errors from the modified design were not always smaller than, but rather similar to or less than, those from the standard design. However, when using pseudo-partial likelihood maximization and IPW estimator, all of the standard errors from the modified design were smaller than those from the standard design. These are consistent with the simulation study in which the design variances of the modified design were smaller than those of the standard design, especially for the IPW approach, which unifies all subsamples and uses individual inclusion probability weights.

## Concluding remarks

We studied the nested case–control sampling, one of the most popular cost-effective epidemiological cohort designs to obtain expensive exposures for a subset of a larger cohort. The standard sampling scheme may include duplicated controls as it replaces previously sampled controls into sampling pools for future selections, which may be inefficient in practice. We investigated the modified sampling scheme that does not replace the sampled controls for future selections so that all controls are unique. We compared the two schemes in estimating relative and baseline hazards for the Cox proportional hazards model, using two approaches: conditional estimation (partial likelihood estimation) and unconditional estimation (inverse probability weighting). For the IPW approach, we also derived the relevant inclusion probabilities for the modified design so that the corresponding weighted estimates are unbiased.

The simulation studies showed that the estimates from the modified sampling are design-unbiased to the full cohort estimates. The bias rather depends on the censoring (more biased with higher censoring) and the number of controls per case (more biased with fewer controls) regardless of the type of NCC designs or the choice of estimation methods. On the other hand, the estimates from the modified sampling mostly had smaller design variance (higher efficiency) compared to the standard design. The variance reduction was more highlighted when the cohort members are less censored and the more controls are matched per case, which indeed increases the odd of re-selecting previously sampled controls for the standard sampling design. In other words, when individuals tend to be excluded from sampling pools due to high censoring and/or when individuals are less likely to be sampled due to sampling few controls per case, the modified design may not improve the standard design that much. It was also remarkable that the IPW estimation was much more improved by the modified design than the conditional estimation. This is because the conditional approach remains the same in the both designs except the components of risk sets, which has little impact on the estimation; however, the IPW method breaks the matching nature and directly makes use of the larger size of unique samples obtained from the modified design.

The NCC design can often be motivated by high computational cost, rather than the high cost of collecting covariate information; for example when huge cohorts are routinely followed up, or when considerably many covariates are of interest. The modified design would also be economically useful even in such circumstances; however, it is recommended to use the conditional estimation method, which gains less efficiency than the IPW method does, because the computation of sampling probabilities and variance estimation of the IPW method might add more computational cost.

## Software

Software in the form of R code, together with a data simulation and complete documentation is available on GitHub: https://github.com/syeeun/nccmod.
